# Clinical Characteristics of Personality and Conduct Disorders in Child Patients With Vasovagal Syncope: A Clinical Case-Control Study

**DOI:** 10.3389/fped.2021.778605

**Published:** 2021-11-26

**Authors:** Tao Wang, Fang Wang, Yifei Li, Xiaoqing Shi, Hongyu Duan, Kaiyu Zhou, Yimin Hua

**Affiliations:** ^1^Department of Pediatric Cardiology, West China Second University Hospital, Sichuan University, Chengdu, China; ^2^Key Laboratory of Birth Defects and Related Diseases of Women and Children, Sichuan University, Ministry of Education, Chengdu, China; ^3^Key Laboratory of Obstetric and Gynecologic and Pediatric Diseases of Sichuan Province, Chengdu, China

**Keywords:** vasovagal syncope, conduct disorders, personality, children, recurrence

## Abstract

**Objective:** To analyze the clinical characteristics of abnormal personality and conduct disorders (CDs) in pediatric patients with vasovagal syncope (VVS).

**Methods:** In this study, we recruited patients diagnosed with VVS at Children's Heart Center from January 2018 to December 2020. Healthy children were recruited as controls. The Eysenck Personality Questionnaire-Child edition (EPQ-C) and Achenbach Child Behavior Checklist (CBCL) were used for the assessment.

**Results:** One hundred and fifty-one VVS patients and 151 healthy controls were included in this study. Compared with the control group, patients in the VVS group had a higher incidence of abnormal personality and were more prone to suffer from CDs. Moreover, pediatric patients with VVS suffered more events of syncope recurrence if they had CDs.

**Conclusion:** Abnormal personality and CDs are common clinical characteristics in pediatric patients with VVS.

## Introduction

Vasovagal syncope (VVS) is the most frequently reported clinical sub-type of neurally mediated syncope (1). Previous clinical analysis demonstrated that VVS is the most common type of syncope in children (2). Vasovagal syncope is classically characterized by specific forms of acute orthostatic intolerance, recurrent syncope, and spontaneous remission (3). Moreover, pediatric patients had the highest incidence of abrupt transient consciousness loss and low postural tone caused by temporary and spontaneously self-terminating global cerebral hypoperfusion (4). The symptoms of VVS usually appear after prolonged standing or sitting and exposure to stress or pain. Although most of the pediatric patients with VVS could have favorable prognosis with less somatic discomfort and cerebral hypoperfusion events after classic treatment, the recurrence rate of VVS still remains high, which was recently reported with a 1-year recurrence rate of 30% (5). At present, classic treatments for pediatric patients with VVS include conventional treatment, peripheral alpha-receptor agonists, and traditional beta-blockers, etc. (6). These treatments experience the bottleneck while further reduction of VVS recurrence.

More and more studies indicated that VVS patients, especially pediatric patients, were more likely to suffer from secondary syncope, which warrants novel therapies (7). However, the underlying mechanisms of secondary syncope are still unclear. On the other hand, latest studies have shown that pediatric patients with VVS, while reaching the adolescence stage, have more mental health problems, including increased bipolar disorder events, uncontrolled antisocial behaviors, and obsessive-compulsive disorders, compared with healthy controls (8). The most common phenomenon for such patients is the poor ability of learning and living, which is associated with VVS-related conduct disorders (CDs) (9).

Conduct disorder is defined as the antisocial and disruptive behavior that violates the rights of others or societal deeds (10). Even though CD was rarely reported in previous VVS clinical reports, increased clinical evidence suggested that CD was a severe and persistent disorder related to cerebrovascular pathological changes or cerebral hypoperfusion (11, 12). In addition, CD could result in personality change, while the latter in pediatric patients with VVS always were mistook for normal phenomenon of adolescent development (13). As for long-term outcomes, CD has been associated with substance use disorders, mental disorders, criminality, and other adverse psychosocial outcomes (14). Intriguingly, all of these CD-related adverse outcomes were also observed in pediatric patients with VVS. As for personality, it is suggested that personality regulates the individual sensitivity to stressors, and in pediatric patients with VVS, emotional arousal, and psychologic uncertainty are the conditions contributing to vasodepressor syncope (15). Recent study has proved that personality characteristics of adult patients with VVS differed significantly compared with healthy control, however, evidence in pediatric patients with VVS is still lacking.

Therefore, our study aimed to explore the clinical characteristics of pediatric patients with VVS.

## Methods

### Study Design and Population

In this prospective study, we enrolled patients who were diagnosed with VVS, based on the 2018 Chinese Pediatric Cardiology Society (CPCS) guidelines, and admitted at the Children's Heart Center, West China Second University Hospital, Sichuan University from January 2018 to December 2020. The inclusion criteria were: (1) school-age children and adolescents with VVS; (2) patients who had VVS associated with predisposing factors, such as prolonged standing, emotional stress, and crowded or stuffy environment; (3) those with a history of syncope; and (4) those with a positive hemodynamic response during the head-up tilt test. The exclusion criteria were: (1) patients with transient loss of consciousness during infancy and early childhood; (2) those with heart diseases, such as cardiomyopathy, pulmonary hypertension, and congenital heart disease; (3) those diagnosed with arrhythmias based on medical history, physical examination, and ECG findings; (4) those with a family history of structural heart diseases or sudden death; and (5) those with syncope caused by other reasons. The healthy children from the same center were recruited as controls during the study period and had normal results during physical examinations. The clinical design and related information were accepted and approved by the Ethics Committee of West China Second University Hospital (No. 2018-002). All guardians of the recruited children have signed the informed consent and fully acknowledged the details of examinations and inspection items.

### Procedure

The pediatric patients with VVS, who were enrolled in the current study, were assessed using Eysenck Personality Questionnaire-Child edition (EPQ-C) and Achenbach Child Behavior Checklist (CBCL). In addition, camera surveillance was performed to determine whether pediatric patients with VVS had disguise propensity. All questionnaires and mental health surveillance were conducted by a trained and qualified pediatrician with a work experience of more than 10 years.

#### EPQ-C

The EPQ-C was performed on the patients to gather the detailed information on personality, including 88 items, for scoring. It was conducted by medical professionals, following the routine procedure. According to the answers (“yes” or “no”) provided by pediatric patients, the questionnaire forms were filled out item by item. Then, based on the requirements of the questionnaire, the scores of the subjects, N (neuroticism or emotional transference), E (introversion and extroversion), P (psychoticism), and L (lie or degree of cover-up), were obtained, and these values were differentiated using the T score table. The corresponding T scores were obtained based on the age of the patient. The normal value of the EPQ-C ranges from 40 to 60. The details of subjects in the questionnaire are given next. E scores: Individuals with higher E scores are more likely to be extroversive with aggressive personality characteristics, including outgoing sociability, crave for stimulation, and tendency to compulsions. Individuals with lower E scores are more likely to be introversive with introverted personality characteristics, including quiet disposition, introspection, and stable emotion. N scores: Individuals with higher N scores suffer from anxiety, worry, and disconsolateness, and react more aggressively upon stimulation. However, individuals with lower N scores react slowly when stimulated and restore their behavior quickly with a higher level of self-control ability. P scores: Individuals with higher P scores have a greater probability to live an insulated life with lesser care for others and more difficulties in adapting to the external environment; whereas, those with lower P scores were more likely to adapt to the external environment with kindness and goodness. L scores: L scores indicated the ability of individuals in covering up the fact. In specific, the L score was positively correlated with incidence of pathological lying. However, latest research indicated that L score could be misled by deliberate avoidance (16). Therefore, we performed mental health surveillance in this study for a better assessment.

#### CBCL

It consists of 113 items and the scores were assessed using three options (none = 0, often = 1, and apparently = 2) for the different subjects. The scores were positively associated with the level of CD. All subjects of CBCL were completed by parents of patients under the guidance of one or two trained clinicians. In our study, nine items of CBCL (depression, discipline violation, dissociative, compulsive, aggressive, bad communication, physical complaints, social withdrawal, and hyperactivity) were regarded as the factors with pivotal effect on diagnosis of CD in pediatric patients with VVS. As reported previously, these factors provided promising reliability and validity on CD diagnosis (17). In addition, one aberrant item in CBCL could confirm CD diagnosis in pediatric patients.

### Outcomes

Mental health surveillance was performed for the objective evaluation of disguise propensity, which could be partly reflected by L items of EPQ-C. All recruited children were monitored under remote sound recorder with electronic watch, and daily conversations were analyzed by medical professionals to determine the disguise propensity according to L items of EPQ-C.

### Statistical Analysis

All results were analyzed using SPSS 20.0 (IBM Corp, Armonk, NY, USA). The Kolmogorov–Smirnov test was used to evaluate whether the continuous data were normally distributed or not. Continuous data with normal distribution were expressed as mean ± standard deviation and were analyzed using the *t*-test. Continuous data with non-normal distribution were presented as median (25th−75th quartile) and were analyzed using the Wilcoxon symbolic rank test. The categorical data were expressed as *n* (%) and were analyzed using Fisher's exact test. The binary logistic regression was used to analyze the correlation between the incidence of VVS in children and EPQ-C and CBCL scores. *P* < 0.05 was considered statistically significant.

## Results

In this study, we assigned 151 pediatric patients who were diagnosed with VVS to the VVS group, and the same number of children were recruited in the control group. Analysis of data showed no differences in baseline information, including gender ratio, age, and body weight, of healthy children and pediatric patients with VVS (*P* > 0.05; Table 1).

**Table 1:** Analysis of baseline data of groups.

**Characteristics**	**VVS (*n* = 151)**	**Control group (*n* = 151)**	***P*-value**
Age (year)	11.08 ± 2.18	11.3 ± 2.14	0.4568
Gender (M/F)			0.4877
Male	71 (47.02%)	65 (43.05%)	
Female	80 (52.98%)	86 (56.95%)	
BMI (kg/cm^2^)	20.5 ± 2.2	20.3 ± 2.3	0.4406

*Continuous data with a normal distribution were expressed as mean ± standard deviation and the categorical data were expressed as n (%)*.

In this study, questionnaire data showed that pediatric patients with VVS had significantly higher incidence of abnormal personality when compared with that of the control group (*P* < 0.05; Table 2). Specifically, in comparison with the control group, the VVS group had higher incidence of introversive personality (normal E scores ≤ 40; 53 [35.10%] vs. 23 [45.10%]) and unstable emotion (normal N scores ≥55; 26 [17.21%] vs. 4 [2.65%]. The CBCL data demonstrated that, when compared with control group, pediatric patients with VVS had significantly higher proportion of CD (*P* < 0.05; Table 2).

**Table 2:** Analysis of EPQ-C scores and CBCL scores of patients.

**Characteristics**	**VVS**	**Control**	***P*-value**
	**(*n* = 151)**	**(*n* = 151)**	
**EPQ-C scores**			
P original score	11.10 ± 5.69	9.19 ± 4.83	0.0011
P standard score	55.77 ± 11.82	51.97 ± 10.38	0.0028
P score ≥60 (ratio)	47/151 (31.13%)	19/151 (12.58%)	<0.0001
E original score	14.86 ± 4.67	16.49 ± 3.55	0.0006
E standard score	41.04 ± 14.05	46.03 ± 11.01	0.0006
E score ≤ 40 (ratio)	53/151 (35.10%)	23/151 (15.23%)	<0.0001
N original score	2.91 ± 2.34	2.13 ± 1.44	0.0006
N standard score	45.51 ± 9.21	42.57 ± 5.46	0.0009
N score ≥55 (ratio)	26/151 (17.21%)	4/151 (2.65%)	<0.0001
L original score	13.47 ± 4.49	14.27 ± 4.25	0.136
L standard score	48.17 ± 10.75	49.97 ± 9.46	0.1491
L score ≥60 (ratio)	12/151 (7.95%)	12/151 (7.95%)	>0.999
**CBCL scores**			
Abnormal social capacity (ratio)	32/151 (21.19%)	18/151 (11.92%)	0.0302
Abnormal behavior activities (ratio)	27/151 (17.88%)	10/151 (6.62%)	0.0028
Communication difficulties (ratio)	22/151 (14.57%)	9/151 (5.96%)	0.0137
Abnormal learning status (ratio)	31/151 (20.52%)	8/151 (5.30%)	<0.0001
Conduct disorder (ratio)	39/151 (25.83%)	15/151 (9.93)	0.0003

*Continuous data with a normal distribution were expressed as mean ± standard deviation, and the categorical data were expressed as n (%)*.

The binary logistic regression was used to evaluate the association between EPQ-C and CBCL scores and VVS in children (Table 3). P standard scores (OR = 1.047, 95% CI = 1.006–1.090, *P* = 0.024) and E standard scores (OR = 0.972, 95% CI = 0.953–0.992, *P* = 0.006) of EPQ-C were found to be independent risk factor for VVS. Moreover, CD of CBCL was related to VVS (OR = 2.457, 95% CI = 1.002–5.907, *P* = 0.045). Further analysis was performed to detect relationship between CD and syncope recurrence in the VVS group. The findings suggest that pediatric patients with VVS suffered more events of syncope recurrence, if they had CD (*P* < 0.05).

**Table 3:** Binary logistic regression analysis for correlation of EPQ-C and CBCL scores with VVS in pediatric patients.

	***P***	**OR**	**95% CI**
Gender	0.506	1.185	0.719	1.952
Age	0.931	1.005	0.898	1.125
N standard score	0.024	1.047	1.006	1.090
E standard score	0.006	0.972	0.953	0.992
P standard score	0.474	1.010	0.983	1.038
L standard score	0.794	1.004	0.976	1.032
Abnormal behavior activities	0.278	0.482	0.129	1.801
Communication difficulties	0.391	0.556	0.146	2.126
Abnormal learning status	0.066	3.766	0.918	15.449
Conduct disorder	0.045	2.457	1.022	5.907

## Discussion

Our study demonstrated that CD was a clinical characteristic for long-term adverse outcome in pediatric patients with VVS. Conduct disorders were significantly positively associated with the rate of syncope recurrence.

Previous studies demonstrated that adult patients with VVS suffered from syncope recurrence due to insufficiency in cerebral blood supply, and VVS-related syncope recurrence caused intense mental stimulation, including terror, tension, anxiety, and other negative emotions (18, 19). In short, this long-term mental distress resulted in CD. Most of the clinical analyses emphasize VVS-induced CD. However, only a few studies aimed to explore whether CD exacerbated the syncope recurrence, and the evidence is scarce, particularly in pediatric patients with VVS (20). Our study demonstrated that pediatric patients with VVS had significant clinical characteristics of CD, including somatization, depression, anxiety, terror, interpersonal tension, and psychosis changes, when compared with healthy children. In addition, our study revealed a high incidence of disguise propensity, which could be an explanation for the negative reports in previous studies and the difficulty of CD diagnosis in pediatric patients with VVS. In terms of personality, pediatric patients with VVS had a higher proportion of introversive and neurotic personality, which resulted in behavior problems.

To our knowledge, our study is the first one to report that CD could exacerbate syncope recurrence in pediatric patients with VVS. We deducted that children could suffer more mental damage under the unhappy experiences of VVS, and clinical studies showed that 21% of pediatric patients with VVS experience fear and uncontrolled worry before syncope. In practice, syncope recurrence is the combined result of physical contributors and mental factors (21). Furthermore, the classic theory proved that physical contributors, such as cerebral hypoperfusion, triggered the syncope, but neglected that mental disorder and CDs could induce syncope recurrence due to psychological maladjustments (22–24). In the field of VVS, CD could be regarded as a state of stress. This stress was caused by VVS and acts back on VVS, playing positive feedback. The underlying mechanism might be that CD deteriorated the stability of autonomic nerves to cause abnormal sympathetic nervous excitement, which was proved previously with the induction of cerebral hypoperfusion (25).

The fear of syncope is another reason for children to suffer with multiple symptoms, including fever, nausea, and dizziness (26). All these unpleasant feelings can cause the body to release syncope-related physiological signals. However, further research on detailed mechanism of VVS is needed for better understanding of the disorder in pediatric patients.

This study has a few limitations. First, the sample size of the study is small. Therefore, the results could not be generalized to a wider population. Another limitation is that interpretation of questionnaires might vary among patients, leading to interpretation bias. Therefore, future studies should be conducted with larger sample size and avoidance of bias for better understanding of the results.

In summary, we have proved that abnormal personality and CDs are clinical characteristics in pediatric patients with VVS.

## Data Availability Statement

The original contributions presented in the study are included in the article/supplementary material, further inquiries can be directed to the corresponding author/s.

## Ethics Statement

The studies involving human participants were reviewed and approved by the Ethics Committee of West China Second University Hospital (approval number: No. 2018-002). Written informed consent to participate in this study was provided by the participants' legal guardian/next of kin.

## Author Contributions

TW was involved in study concept, study design, and manuscript preparation. FW carried out the definition of intellectual content and manuscript review. YL handled data analysis and statistical analysis. XS carried out data acquisition. HD conducted the clinical studies. KZ was involved in the literature research and manuscript editing. YH performed the role of guarantor for the integrity of the entire study. All authors have read and approved this article.

## Funding

This article was supported by the National Natural Science Foundation of China (No 81701888), Science and Technology Program of Sichuan (2019YFS0239), and Health and Family Planning Commission Foundation of Sichuan Province (No. 17PJ262).

## Conflict of Interest

The authors declare that the research was conducted in the absence of any commercial or financial relationships that could be construed as a potential conflict of interest.

## Publisher's Note

All claims expressed in this article are solely those of the authors and do not necessarily represent those of their affiliated organizations, or those of the publisher, the editors and the reviewers. Any product that may be evaluated in this article, or claim that may be made by its manufacturer, is not guaranteed or endorsed by the publisher.
